# Mice with different susceptibility to tick-borne encephalitis virus infection show selective neutralizing antibody response and inflammatory reaction in the central nervous system

**DOI:** 10.1186/1742-2094-10-77

**Published:** 2013-06-27

**Authors:** Martin Palus, Jarmila Vojtíšková, Jiří Salát, Jan Kopecký, Libor Grubhoffer, Marie Lipoldová, Peter Demant, Daniel Růžek

**Affiliations:** 1 Institute of Parasitology, Biology Centre of the Czech Academy of Sciences, Branišovská 31, České Budějovice CZ-37005, Czech Republic; 2 Faculty of Science, University of South Bohemia, Branišovská 31, České Budějovice CZ-37005, Czech Republic; 3 Institute of Molecular Genetics, Academy of Sciences of the Czech Republic, Vídeňská 1083, Prague CZ-14220, Czech Republic; 4 Department of Virology, Veterinary Research Institute, Hudcova 70, Brno CZ-62100, Czech Republic; 5 Roswell Park Cancer Institute, Elm and Carlton Streets, Buffalo, New York 14263, USA

**Keywords:** Tick-borne encephalitis, Flavivirus encephalitis, Neuroinflammation, Antibody production

## Abstract

**Background:**

The clinical course of tick-borne encephalitis (TBE), a disease caused by TBE virus, ranges from asymptomatic or mild influenza-like infection to severe debilitating encephalitis or encephalomyelitis. Despite the medical importance of this disease, some crucial steps in the development of encephalitis remain poorly understood. In particular, the basis of the disease severity is largely unknown.

**Methods:**

TBE virus growth, neutralizing antibody response, key cytokine and chemokine mRNA production and changes in mRNA levels of cell surface markers of immunocompetent cells in brain were measured in mice with different susceptibilities to TBE virus infection.

**Results:**

An animal model of TBE based on BALB/c-c-STS/A (CcS/Dem) recombinant congenic mouse strains showing different severities of the infection in relation to the host genetic background was developed. After subcutaneous inoculation of TBE virus, BALB/c mice showed medium susceptibility to the infection, STS mice were resistant, and CcS-11 mice were highly susceptible. The resistant STS mice showed lower and delayed viremia, lower virus production in the brain and low cytokine/chemokine mRNA production, but had a strong neutralizing antibody response. The most sensitive strain (CcS-11) failed in production of neutralizing antibodies, but exhibited strong cytokine/chemokine mRNA production in the brain. After intracerebral inoculation, all mouse strains were sensitive to the infection and had similar virus production in the brain, but STS mice survived significantly longer than CcS-11 mice. These two strains also differed in the expression of key cytokines/chemokines, particularly interferon gamma-induced protein 10 (IP-10/CXCL10) and monocyte chemotactic protein-1 (MCP-1/CCL2) in the brain.

**Conclusions:**

Our data indicate that the genetic control is an important factor influencing the clinical course of TBE. High neutralizing antibody response might be crucial for preventing host fatality, but high expression of various cytokines/chemokines during TBE can mediate immunopathology and be associated with more severe course of the infection and increased fatality.

## Background

Flaviviruses, a group of small, enveloped, positive-sense, single-stranded RNA viruses, include several medically very important pathogens. Especially Japanese encephalitis virus, yellow fever virus, West Nile virus, dengue virus, Murray Valley encephalitis virus and tick-borne encephalitis virus (TBEV) are responsible for large outbreaks of fatal encephalitis or hemorrhagic fevers at diverse geographical regions around the world. Tick-borne encephalitis (TBE), a disease caused by TBEV, represents one of the most important and serious neuroinfections in Europe and northeastern Asia. More than 13,000 clinical cases of TBE, including numerous deaths, are reported annually. Despite the medical importance of this disease, some crucial steps in the development of encephalitis remain poorly understood. In humans, TBEV may produce a variety of clinical symptoms, from an asymptomatic disease (70-90% of cases) to a fever and acute or chronic progressive encephalitis. This is influenced by a variety of factors, e.g., the inoculation dose and virulence of the virus [1], the age, sex and immune status of the host [2], and also susceptibility based on the host’s genetic background. Studies of animal models and epidemiological studies in humans have shown that many apparently non-hereditary diseases, including infectious diseases, develop predominantly in genetically predisposed individuals and that this predisposition is caused by multiple genes [3]. In humans, a functional Toll-like receptor 3 gene may be a risk factor for TBEV infection [4]. A deletion within the chemokine receptor CCR5 (CCR5Δ32), which plays an important role in leukocyte transmigration across the blood–brain barrier, is significantly more frequent in patients with TBE than in TBE-naïve patients with aseptic meningitis [5]. Moreover, the severity and outcome of TBE is associated with variability in the 2'-5'-oligoadenylate synthetase gene cluster (family members are interferon-induced antiviral proteins that play an important role in the endogenous antiviral pathway) [6] and with the rs2287886 single nucleotide polymorphism located in the promoter region of the human *CD209* gene [7]. This gene encodes dendritic cell-specific ICAM3-grabbing nonintegrin (DC-SIGN), a C-type lectin pathogen-recognition receptor expressed on the surface of dendritic cells and some types of macrophages [7]. Taken together, polymorphism in various genes may largely influence the sensitivity of the host to the infection and determine the severity of this disease.

While in humans involvement of genetic factors in the control of the susceptibility to TBEV infection is quite difficult to investigate, mice provide a useful small animal model for such a kind of study [8]. Mice are suitable animal models of infection with TBEV because they can reproduce symptoms and physiopathological markers as observed in severe cases in humans. A high susceptibility of most laboratory mouse strains to flavivirus infection has been genetically mapped to a stop codon mutation in the coding region of the 2´-5´-oligoadenylate synthetase gene *Oas 1b*[9].

In our study, we developed an animal model of TBE showing several manifestations of the disease in relation to the genetic background, which is not based on the previously published mutation in the *Oas 1b* gene. We analyzed the sensitivity to TBEV in CcS/Dem (CcS) recombinant congenic (RC) strains of mice [10] derived from the background strain BALB/cHeA (BALB/c) and the donor strain STS. Each CcS strain contains a unique random set of about 12.5% genes from the donor strain STS and 87.5% genes from the background strain BALB/c [10]. This system has been very useful in research of bacterial [11] and parasitic [12-18] diseases, as well as in cancer [19-23].

In this study, we identified mouse strains that exhibit high, intermediate and low sensitivity to TBEV infection. Virus growth, key cytokine and chemokine mRNA production in the brain and neutralizing antibody response were measured. Our data suggest that the genetic control represents one of the important factors that influence the clinical course of TBE and that also other genes than the previously described *Oas 1b* are involved in the determination of host susceptibility to the infection. While high neutralizing antibody response might be crucial for preventing host fatality, high local expression of various proinflammatory cytokines/chemokines in the brain during TBE can be associated with a more severe course of the infection and higher fatality. Our data may be instrumental in the development of future therapeutic strategies aimed at treating or preventing TBE neuropathogenesis.

## Material and methods

### Mice

**Figure 1:**
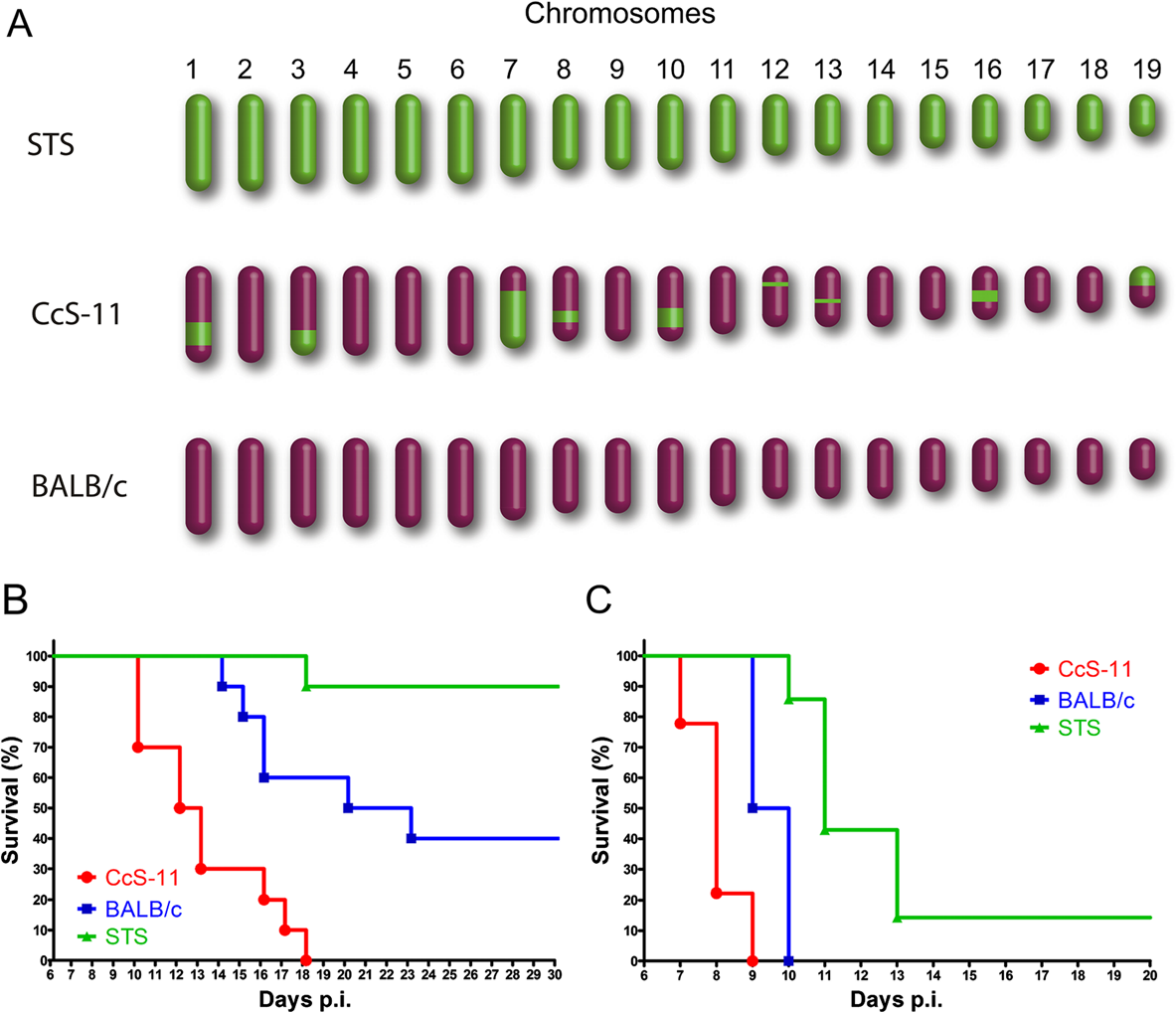
**Chromosomal composition of the mouse strains used in this study based on precise chromosomal mapping.** The RC strain CcS-11 contains a random set of ~12.5% genes from strain STS donor strain and 87.5% genes from strain BALB/c (background strain). Sex chromosomes are not shown in this figure (**A**). Survival of mice after s.c. inoculation of TBEV. Mice (*n* = 10 per group) were inoculated with 10^3^ pfu of TBEV and observed for lethality (**B**). Survival of mice (BALB/c, *n* = 8; CcS-11, *n* = 9; STS, *n* = 7) after i.c. inoculation of 10 pfu of TBEV (**C**).

Specific pathogen-free mice of parental strains BALB/c, STS and ten randomly selected RC strains (see below) were used in the experiments. RC strains were in more than 90 generation of inbreeding and therefore highly homozygous. Genetic composition of the strain CcS-11 is schematically shown in Figure 1A. Sterilized pellet diet and water were supplied ad libitum. In all experiments, female mice aged 12–15 weeks at the time of infection were used. The mice were housed in plastic cages with wood-chip bedding, situated in a specific pathogen-free room with a constant temperature of 22°C and a relative humidity of 65%. The research complied with all relevant European Union guidelines for work with animals and was in accordance with the Czech national law and guidelines on the use of experimental animals and protection of animals against cruelty (Animal Welfare Act no. 246/1992 Coll.). The protocol was approved by the Committee on the Ethics of Animal Experiments of the Institute of Parasitology and of the Departmental Expert Committee for the Approval of Projects of Experiments on Animals of the Academy of Sciences of the Czech Republic (permit no. 165/2010).

### Virus infection

All experiments were performed with European prototypic TBEV strain Neudoerfl (a generous gift from Prof. F.X. Heinz, Medical University of Vienna). The virus was originally isolated from the tick *Ixodes ricinus* in Austria in 1971 and has been extensively characterized including its complete genome sequence and the determination of the three-dimensional structure of its envelope protein [24]. This strain was passaged four times in brains of suckling mice.

Mice were infected either subcutaneously (s.c.) into the scruff of the neck with 10^3^ pfu or intracerebrally (i.c.) with 10 pfu of the virus. Mice were scored for mortality for a period of 28 days post-infection (p.i.), and survival curves were established.

### Plaque assay

The virus titers were determined by plaque assay on porcine kidney stable cell (PS) monolayers under a carboxymethyl-cellulose overlay, as described previously [25]. Infectivity was expressed as plaque-forming units (pfu) per ml (or g of brain tissue).

### Virus growth in mice

At the given time point p.i., three mice of each group were anesthetized and killed by cervical dislocation. Specimens of blood serum and brains were collected. Brains were individually weighed and prepared as 20% suspensions in phosphate-buffered saline (PBS) using TissueLyser II (Qiagen). The homogenate was clarified by centrifugation at 14,000× g for 10 min at 4°C, and supernatant fluids were titrated by plaque assay on PS cells. The detection thresholds were 1.4 log_10_ pfu/ml for serum and 2.1 log_10_ pfu/g for brain suspensions.

### Real-time quantitative RT-PCR

Real-time quantitative PCR was performed by the TaqMan Gene Expression Assays (Applied Biosystems) as previously described [26]. RNA was isolated from the brain tissue pellets using RNeasy Mini Kit (Qiagen), according to the recommendations of the manufacturer. cDNA was synthesized using a High Capacity RNA-to-cDNA Kit (Applied Biosystems), according to the manufacturer's protocol. The synthesized cDNAs were used as templates for real-time PCR. In quantitative RT-PCR the following primer probe sets from Applied Biosystems were used: TNFα (Mm00443258_m1), IFNγ (Mm 01168134_m1), CCL2/MCP1 (Mm 00441242_m1), CCL3/MIP1α (Mm00441258_m1), CCL4/MIP1β (Mm00443111_m1), CCL5/RANTES (Mm01302428_m1), CD4 (Mm00442754_m1), CD8b1 (Mm00438116_m1), CD11β (Mm00434455_m1), CD19 (Mm00515420_m1), IL1a (Mm00439620_m1), IL1β (Mm01336189_m1), IL6 (Mm00446190_m1), IL2 (Mm00434256_m1), IL4 (Mm00445259_m1), IL5 (Mm00439646_m1), IL10 (Mm00439614_m1) and IP10/CXCL10 (Mm00445235_m1). Mouse beta actin was used as a housekeeping gene. Amplification conditions were: 2 min at 50°C; 10 min at 95°C; 40 cycles of denaturation at 95°C for 15 s and annealing/extension at 60°C for 1 min.

Quantification of gene expression was performed using the comparative CT method and reported as the fold difference relative to the housekeeping gene. To calculate the fold change, the CT of the housekeeping gene was subtracted from the CT of the target gene to yield the ΔCT. Change in expression of the normalized target gene was expressed as 2^-ΔΔCT^ where ΔΔCT = ΔCT samples - ΔCT controls as previously described [27].

### Plaque reduction neutralization test

The titers of neutralizing antibodies against TBEV in mouse sera were determined by the plaque reduction neutralization test (PRNT) as described by Bárdoš [28] with slight modifications. Sera (including positive and negative controls) were diluted 1:4 in L-15 medium (Leibowitz; Sigma) supplemented with 1% antibiotics (penicillin, streptomycin, amphotericin B; Sigma) and 3% inactivated newborn calf serum. After inactivation of the virus and complement in the sera by heating at 56°C for 30 min, two-fold serial dilutions of the samples in L-15 medium were incubated with 10^3^ pfu of TBEV (the virus test dose was adjusted so that it caused almost confluent plaques, 90-95% cytolysis) for 90 min at 37°C; 5 × 10^4^ PS cells were added to each well. After 6 days of incubation, the cell supernatant was removed and cells were fixed and stained as described previously [25]. The last dilution of serum that caused an 80-100% reduction in cytolysis of the virus test dose was regarded as the serum titer.

### Statistical analysis

The differences in survival time of infected mice were analyzed by survival analysis (log-rank Mantel-Cox test). All other data were analyzed by one-way ANOVA (Newman-Keuls multiple comparison test). Data without normal distribution were transformed by use of the X’ = ln(X) formula. All analyses were performed using GraphPad Prism 5.00 (GraphPad Software, Inc., USA); *p*-values < 0.05 were considered significant.

## Results

### CcS/Dem RC mouse strains show diverse susceptibilities to infection with TBEV

To study the susceptibility of mice to TBEV, we infected females of the strains BALB/c, STS and RC strains CcS-3, CcS-7, CcS-9, CcS-11, CcS-15 and CcS-16 s.c. with 10^4^ pfu of TBEV, and survival was recorded. Mice with different genotypes exhibited different susceptibilities to TBEV (Additional file 1: Figure S1). The most striking differences in survival rates and mean survival times were seen in parental strains BALB/c and STS, and RC strain CcS-11 (Figure 1B). CcS-11 mice were shown to be most susceptible, while STS mice are the least susceptible. BALB/c mice exhibited intermediate susceptibility. Mean survival time (MST) of the CcS-11 mice was 13.1 ± 3 days, BALB/c 22.8 ± 7.5 days and STS 29.7 ± 4.1 days (*p* < 0.05). Differences were also found in case of survival rates. While all CcS-11 mice died following the infection, 40% of infected BALB/c and 90% of STS mice survived until the end of the experiment (Figure 1B). STS mice remained free of any clinical signs of the disease during the whole experiment (including that one mice that died); BALB/c mice exhibited signs of rough fur, hunching or back limb paralysis. Some CcS-11 mice had similar signs as BALB/c mice, but some died without previous appearance of any kind of signs.

In order to test the susceptibility to the TBEV infection if host factors before viral entry of the CNS are avoided, we infected females of the strains BALB/c, STS and RC strains CcS-3, CcS-4, CcS-5, CcS-7, CcS-9, CcS-11, CcS-12, CcS-15, CcS-18 and CcS-20 i.c. with 10 pfu of TBEV. In this case, all mouse strains were susceptible to the infection. Most strains died almost at the same time p.i., i.e., between days 10 and 11 p.i. (Additional file 1: Figure S2). However, differences were seen in the MST in case of CcS-11 (MST of 8.0 ± 0.7 days), BALB/c (MST of 9.5 ± 0.5 days) and STS strains (MST of 12.4 ± 2.7 days) (*p* < 0.05; Figure 1C). Therefore, also factors acting directly in the CNS of the infected mice may influence the survival time. For all subsequent experiments, mice of strains CcS-11, BALB/c and STS were selected as representatives of high-, medium- and low-susceptible hosts.

### Virus load in serum and brain of TBEV-infected mice

**Figure 2:**
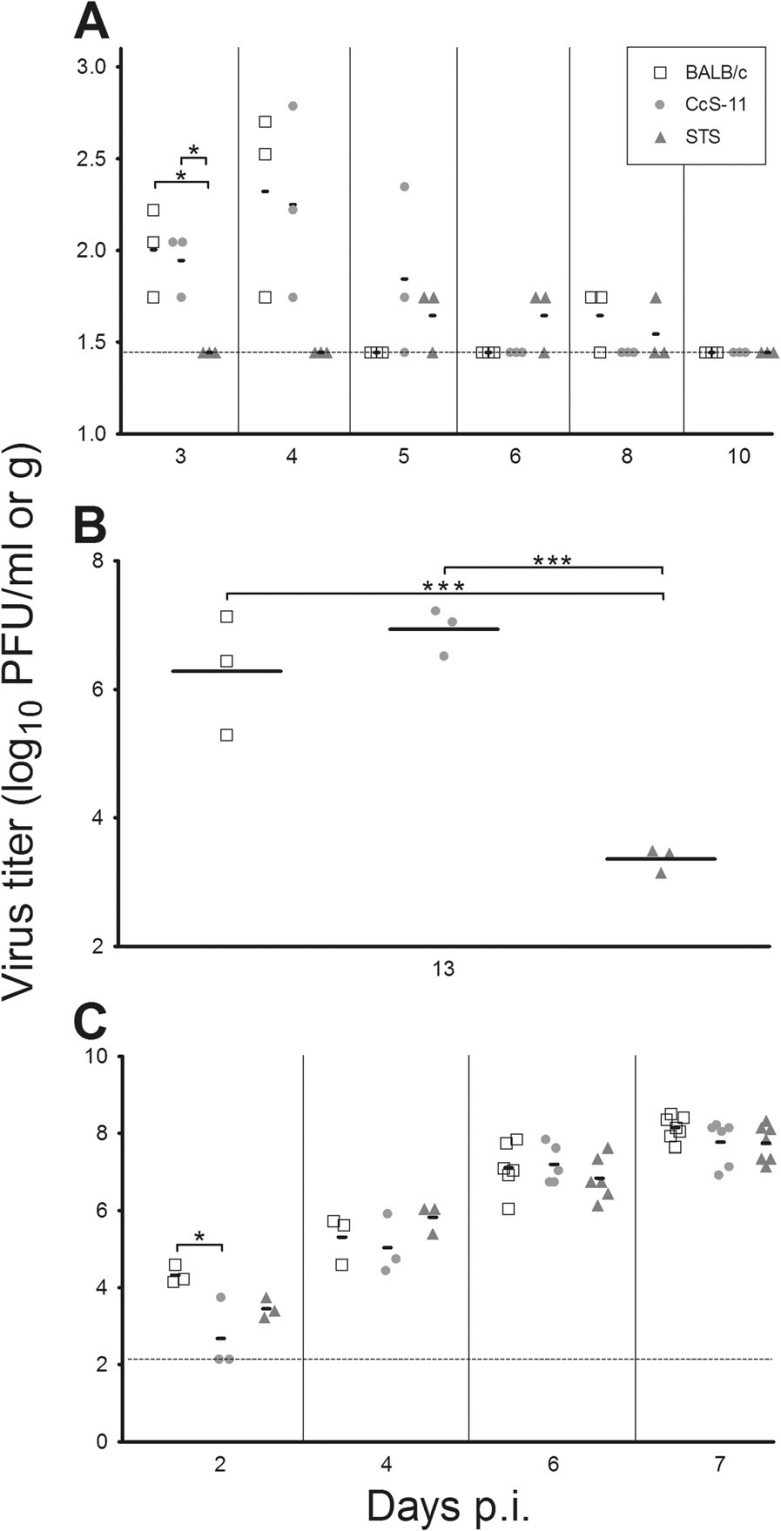
**Viral loads in sera of BALB/c, CcS-11 and STS mice after s.c. inoculation of 10**^**3 **^**pfu of TBEV.** Virus titer was determined by plaque assay on PS cells (**A**). Virus titer in brains of BALB/c, CcS-11 and STS mice after s.c. inoculation was assayed on day 13, i.e., at the time of clinically manifest neuroinfection (**B**). Virus titer in brains of BALB/c, CcS-11 and STS mice after i.c. inoculation of 10 pfu of TBEV (**C**). The detection thresholds were 1.4 log_10_ pfu/ml for serum and 2.1 log_10_ pfu/g for brain suspensions.

Serum samples of BALB/c, CcS-11 and STS mice were harvested for titration at various time points after s.c. inoculation of TBEV. The virus titers in individual samples were determined by plaque assay on PS cells. While the peak of viremia was observed in BALB/c and CcS-11 mice reaching 2–2.5 log_10_ pfu/ml at days 3 and 4 p.i., no virus was detected at the same time points in STS mice (*p* < 0.05). STS mice had only traces of the virus in serum (<2 log_10_ pfu/ml) at later time intervals, i.e. at day 5, 6 and 8 p.i. (Figure 2A).

Virus load in the brain after s.c. inoculation was assayed at day 13 p.i., i.e. at the time point when most of the CcS-11 and BALB/c mice exhibited neurological signs of the infection (Figure 2B). No difference was found in the virus titer in brain of the infected BALB/c and CcS-11 mice (titer reaching 6–7 log_10_ pfu/g), but a much lower titer was observed in brains of infected STS mice (approximately 3 log_10_ pfu/g; *p* < 0.005).

After i.c. inoculation, a dynamics of virus growth in the brain was investigated. Increasing virus titers were seen before mice showed signs of the infection. The virus growth was almost identical in all three mouse strains, reaching its maximum at day 7 (8 log_10_ pfu/g) (Figure 2C). The only difference was observed at day 2, when BALB/c mice had slightly higher virus titers in the brain than CcS-11 mice (*p* < 0.05).

### Immune cell accumulation in the CNS of TBEV infected mice

Accumulation of immunocompetent cells in the brain of the infected BALB/c, STS and BALB/c mice was quantified as the changes in mRNA levels of cell surface markers of B-cells (CD19), T-helper cells (CD4), cytotoxic T-cells (CD8β) and macrophage/monocyte/granulocyte/NK cells (CD11b).

**Figure 3:**
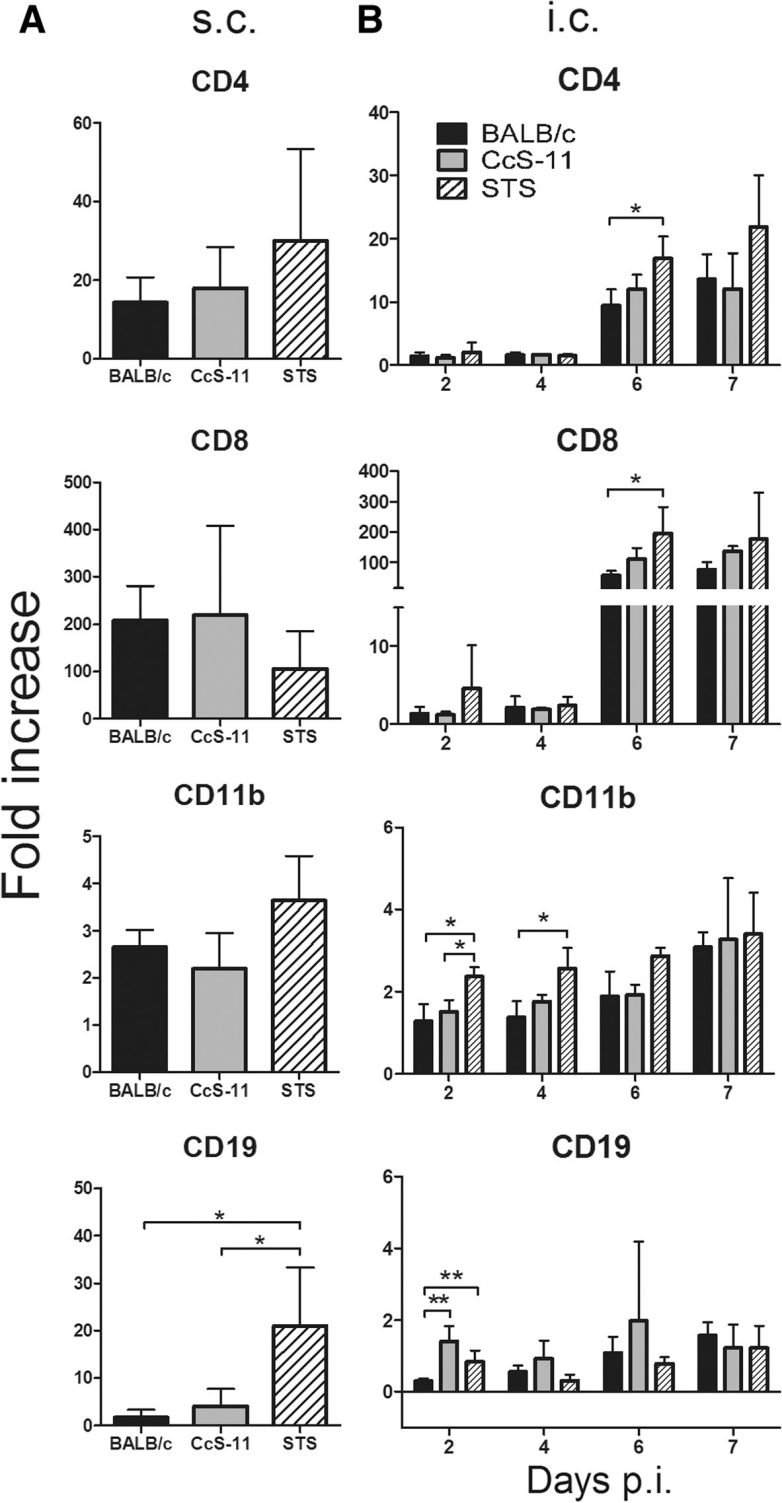
**Immune/inflammatory cell accumulation in brains of BALB/c, CcS-11 and STS mice after s.c. (13 day p.i.) (A) or i.c. (B) inoculation of TBEV.** Immune cell accumulation in the CNS was quantified as the changes in mRNA levels of cell surface markers of B-cells (CD19), T-helper cells (CD4), cytotoxic T-cells (CD8β) and macrophage/monocyte/granulocyte/NK cells (CD11b), and normalized to expression of housekeeping gene, mouse beta actin. Data are expressed as the mean + standard deviation of the fold increase in the level of the mRNA in comparison to uninfected control mice. *p < 0.05; **p < 0.005 (*n* = 3 mice per group and experiment).

In case of s.c. inoculation of TBEV (Figure 3A), the immune cell antigen mRNA accumulation was assayed at day 13 p.i. During our preliminary experiments, mice were also sampled and assayed at days 8 and 10 p.i. (data not shown). However, at these time points only some mice had virus in their CNS, making the results too variable. The day 13 p.i. was the first interval when all mice had virus in their brains. CD4, CD8β and CD11b mRNA levels in the brains of infected mice at day 13 p.i. became significantly higher than those of normal uninfected mice at day 13. p.i. However, CD19 mRNA levels did not significantly increase in BALB/c and CcS-11 mice, but became elevated in STS mice (*p* < 0.05).

After i.c. inoculation of TBEV (Figure 3B), the dynamics of immune cell accumulation in the CNS was investigated at days 2, 4, 6 and 7 p.i. No significant increase in the CD19 mRNA levels was seen in any of the investigated mouse strain at 4, 6 and 7 days p.i. Levels of CD11b mRNA increased in brains of STS mice as early as at day 2 and 4 p.i., whereas no increase was observed in BALB/c and CcS-11 mice until day 4 p.i. From day 6, a slight but significant increase of CD11b levels was observed in all mice tested; however, there was no difference between the individual mouse strains. CD4 and CD8β mRNA levels increased remarkably (10-20-fold increase of CD4 mRNA and 100-200-fold increase of CD8β mRNA compared to negative controls) in all investigated mouse strains from day 6 p.i. Higher levels of CD4 as well as CD8β mRNA levels were detected in brains of infected STS than BALB/c mice at day 6 p.i. (*p* < 0.05).

### Expression of key inflammatory cytokines/chemokines in brains of TBEV infected mice

**Figure 4:**
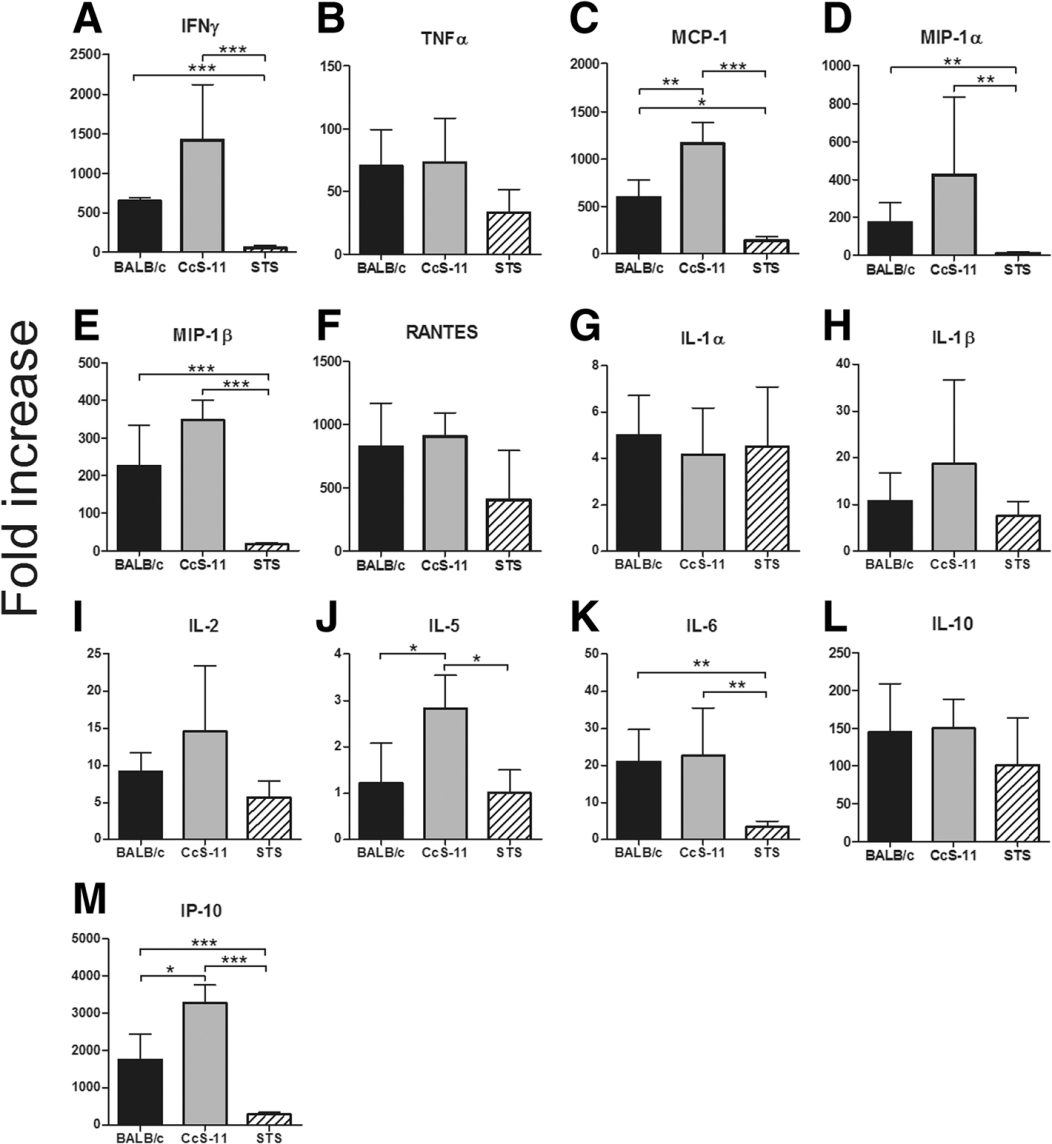
**Cytokines and chemokines produced in brains of BALB/c, CcS-11 and STS mice inoculated s.c. with 10**^**3 **^**pfu of TBEV.** The brains were collected and cytokine production assayed on day 13 p.i. The levels of cytokine/chemokine mRNA [IFNγ (**A**); TNFα (**B**); MCP-1 (**C**); MIP-1α (**D**); MIP-1β (**E**); RANTES (**F**); IL-1α (**G**); IL-1β (**H**); IL-2 (**I**); IL-5 (**J**); IL-6 (**K**); IL-10 (**L**) and IP-10 (**M**)] were quantified by real-time RT-PCR and standardized to the mRNA levels of the housekeeping gene, beta actin. Data are expressed as the mean + standard deviation of the fold increase in the level of the mRNA in comparison to uninfected control mice. *p < 0.05; **p < 0.005, ***p < 0.0005. (*n* = 3 mice per group and experiment).

**Figure 5:**
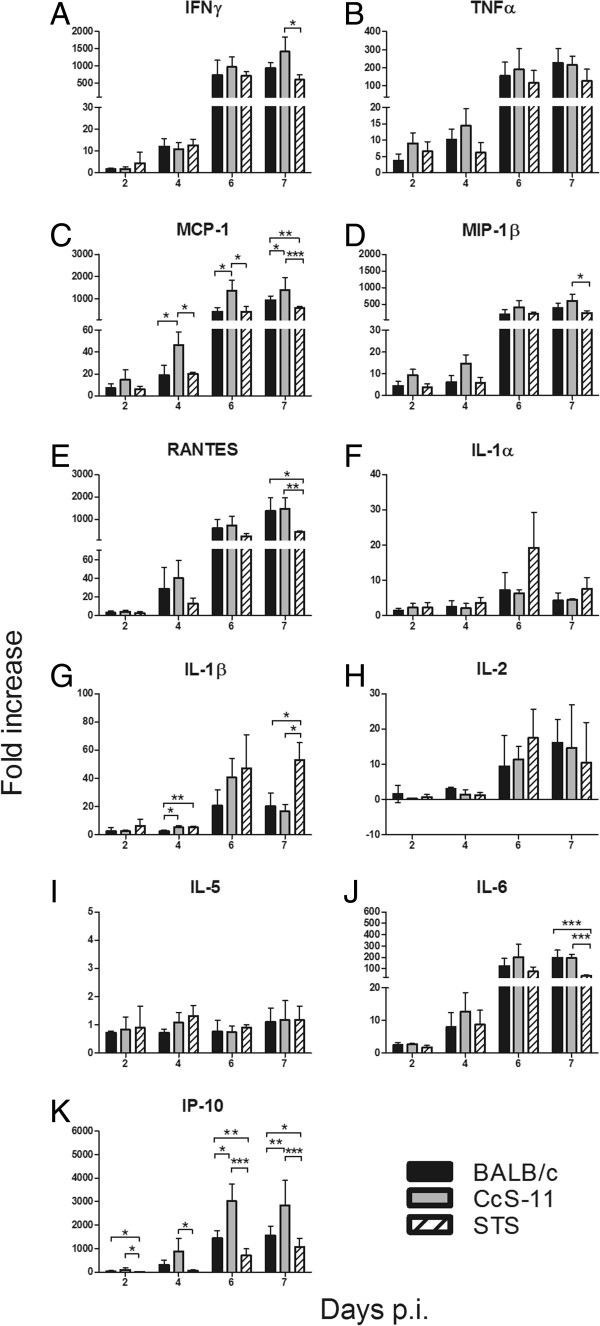
**Cytokines and chemokines produced in brains of BALB/c, CcS-11 and STS mice inoculated i.c. with 10 pfu of TBEV.** The levels of cytokine/chemokine mRNA [IFNγ (**A**); TNFα (**B**); MCP-1 (**C**); MIP-1β (**D**); RANTES (**E**); IL-1α (**F**); IL-1β (**G**); IL-2 (**H**); IL-5 (**I**); IL-6 (**J**); and IP-10 (**K**)] were quantified by real-time RT-PCR and standardized to the mRNA levels of the housekeeping gene, beta actin. Data are expressed as the mean + standard deviation of the fold increase in the level of the mRNA in comparison to uninfected control mice. *p < 0.05; **p < 0.005, ***p < 0.0005 (*n* = 3 mice per group and experiment).

The levels of mRNA specific for a broad range of cytokines (TNF-α, IFNγ, IL-1α, IL-1β, IL-2, IL-4, IL-5, IL-6 and IL-10) and chemokines (CCL2 (chemokine ligand 2)/MCP-1 (monocyte chemotactic protein-1), CCL3/MIP-1α (macrophage inflammatory protein-1α), CCL4/MIP-1β (macrophage inflammatory protein-1β), CCL5/RANTES (regulated upon activation, normal T-cell expressed and secreted) and interferon-γ-inducible protein-10 (IP-10)/CXCL/10) were quantified in brains of TBEV infected BALB/c, CcS-11 and STS mice by real-time RT-PCR, and standardized to the levels of the housekeeping gene, beta actin, mRNA (Figures 4 and 5).

After the s.c. inoculation of TBEV (Figure 4), the levels of cytokine/chemokine mRNA were measured on day 13 p.i. During our preliminary experiments, mice were also sampled and assayed at days 8 and 10 p.i. (data not shown). However, at these time points only some mice had virus in their CNS, making the results too variable. The day 13 p.i. was the first interval when all mice had virus in their brains. Significantly increased mRNA levels of TNF-α, IFNγ, IL-1α, IL-1β, IL-2, IL-6, IL-10, MCP-1, RANTES and IP-10 were seen in the TBEV-infected mice of all strains when compared to negative uninfected controls at day 13 p.i. In case of CCL3/MIP-1α (Figure 4D) and CCL4/MIP-1β (Figure 4E) mRNA, only sensitive strains BALB/c and CcS-11 exhibited upregulated expression after the TBEV infection, whilst the expression in infected STS mice was not changed. The levels of IL-5 mRNA were increased only in brains of infected CcS-11 mice (Figure 4J), but the infection did not affect the expression of this cytokine in brains of BALB/c and STS mice. The levels of IFN-γ were the highest in brains of infected CcS-11 mice; BALB/c mice exhibited higher levels than STS (*p* < 0.0005; Figure 4A). The levels of upregulation of IL-6 mRNA expression were similar in BALB/c and CcS-11 mice, but both these strains exhibited significantly enhanced expression in comparison to STS mice (*p* < 0.005; Figure 4K).

Major differences in the cytokine mRNA expression among the infected BALB/c, CcS-11 and STS mice were seen in case of MCP-1 (Figure 4C) and IP-10 (Figure 4M) mRNA. CcS-11 mice had the highest levels of MCP-1 and IP-10 mRNA in brains, while BALB/c had lower levels of MCP-1 and IP-10 mRNA when compared to CcS-11 mice, but higher when compared to STS mice (Figure 4C, M).

The dynamics of the changes in cytokine/chemokine mRNA expression was investigated at days 2, 4, 6 and 7 after i.c. inoculation of TBEV (Figure 5). No changes in the levels of IL-5 mRNA (Figure 5I) were observed in any of the studied mouse strains at any time point. TNF-α (Figure 5B), CCL2/MCP-1 (Figure 5C) and CCL4/MIP-1β (Figure 5D) mRNA levels increased significantly from day 2 p.i. A significant increase in the level of mRNA for IFN-γ, IL-1β, IL-2, IL-6 and CCL5/RANTES (Figure 5 A, G, H, J, E) was seen from day 4 p.i. Expression of IL-1α mRNA was enhanced from the day 6 p.i. (Figure 5F). A large variation in the values of MIP-1α, IL-10 (data not shown) and IL-2 (Figure 5H) mRNA was observed between the individual mice within the strain each day after the i.c. inoculation of TBEV. IFN-γ mRNA levels were much higher in CcS-11 mice at day 7 p.i. when compared to STS mice (*p* < 0.05). Similarly, the lowest levels of IL-6 (*p* < 0.0005) and CCL5/RANTES (BALB/c vs. STS, *p* < 0.05; CcS-11 vs. STS, *p* < 0.005) mRNA were detected in STS mice versus BALB/c and CcS-11 mice at day 7 p.i. In contrast, STS mice had very enhanced levels of IL-1β mRNA at day 7 p.i. in comparison to BALB/c and CcS-11 mice (*p* < 0.05). The greatest differences among the infected BALB/c, CcS-11 and STS mice were seen in case of MCP-1 and IP-10 mRNA. CcS-11 mice had the highest levels of MCP-1 mRNA in brains at days 4, 6 (*p* < 0.05) and 7 p.i. (BALB/c vs. STS, *p* < 0.005; CcS-11 vs. BALB/c, *p* < 0.05; CcS-11 vs. STS, *p* < 0.0005). The levels of IP-10 mRNA were the highest in CcS-11 mice at all investigated time points p.i., the highest differences being observed at day 6 p.i. (BALB/c vs. CcS-11 and STS vs. CcS-11, *p* < 0.005) and at day 7 p.i. (BALB/c vs. CcS-11, *p* < 0.005, and STS vs. CcS-11, *p* < 0.0005). BALB/c had lower levels of IP-10 mRNA than CcS-11 mice (*p* < 0.05), but higher than STS mice (*p* < 0.05). A correlation between the IP-10 mRNA levels with the sensitivity of mice to the TBEV infection was observed (Figures 1C, 5K). No changes in the expression of IL-4 mRNA were seen in any mouse strain after either s.c. or i.c. TBEV inoculation (data not shown).

### TBEV-specific antibodies elicited in infected mice

**Figure 6:**
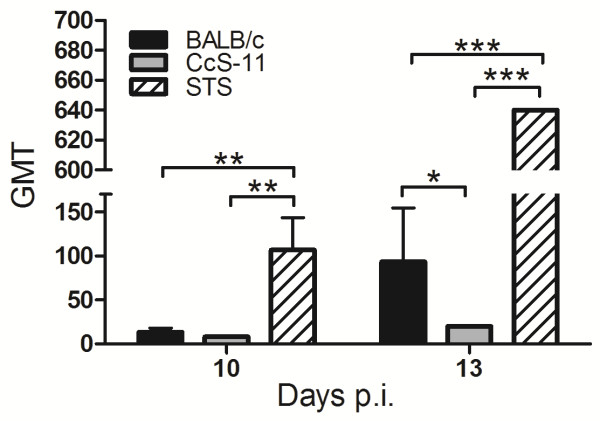
**TBEV-specific antibody responses in BALB/c, CcS-11 and STS mouse sera (*n *= 3 mice per group) after s.c. inoculation of TBEV.** The plaque reduction neutralization test in PS cells was used to quantitate anti-TBEV neutralizing antibodies. Data are expressed as the geometric mean titer (GMT) + standard deviation. *p < 0.05; **p < 0.005, ***p < 0.0005.

In order to compare the host’s humoral immune response in the infected BALB/c, CcS-11 and STS mice after s.c. inoculation of TBEV, TBEV-specific antibodies were tested at days 5, 8, 10 and 13 p.i.. The titer of neutralizing antibodies was determined by PRNT (plaque reduction neutralization test) assay. Before day 10, a very low titer of neutralizing antibodies and no difference between the strains were observed (data not shown). At day 10 p.i., very low titers of neutralizing antibodies were found in serum of BALB/c and CcS-11 mice (less than 1:20), but STS mice exhibited neutralizing titers of 1:100 (*p* < 0.005). At day 13 p.i., no increase in the titer of neutralizing antibodies was seen in CcS-11 mice. BALB/c mice exhibited neutralizing titers of 1:100, but STS mice had titers reaching 1:640 (*p* < 0.0005) (Figure 6). No neutralizing activity was detected in sera from control uninfected mice.

## Discussion

### Host genotype as an important determinant of susceptibility to TBEV infection

Mice provide a useful tool for study of human TBE, since they recapitulate the pathological and pathophysiological processes seen in severe human TBE cases. However, biological and virological markers indicating the severity of TBE in mice as well as in humans remain unclear. The severity of TBE can be influenced by a variety of factors, e.g., the inoculation dose and virulence of the virus [1], the age, sex and immune status of the host [2], and susceptibility based on the host’s genetic background. Generally, age of the host is the most common host risk factor identified for severe forms of flavivirus encephalitis [29]. However, genetic host risk factors play an important role in the development of severe flaviviral neurological diseases as well. Several genetic host factors associated with the severe forms of flavivirus encephalitis have been identified. These include polymorphisms in *HLA-A* and *HLA-B*, polymorphism located in the promoter region of the human *CD209* gene [7] and functional Toll-like receptor 3 gene [4], deletion in *CCR5*[5] and variability in 2´-5´-oligoadenylate synthetase gene cluster [6]. In our study, we developed a new mouse model of hosts differing in the susceptibility to TBEV infection based on CcS/Dem RC mouse strains. We observed clearly different patterns in the susceptibility of the CcS/Dem RC strains to s.c. infection with TBEV. Out of the eight strains tested, one (STS) exhibited low susceptibility, five intermediate susceptibility (CcS-3, CcS-7, CcS-9, CcS-16 and BALB/c) and two strains (CcS-11 and CcS-15) high susceptibility (Additional file 1: Figure S1). Differences in MST between the strains CcS-11, BALB/c and STS were also observed after i.c. inoculation of TBEV, i.e., CcS-11 mice had the shortest MST, BALB/c medium MST and STS the longest MST and higher survival (Figure 1C). These differences indicate the presence of TBEV susceptibility genes. Different susceptibility to virus infection in recombinant inbred strains between BALB/c and STS mice was also observed in case of infection with murine coronavirus [30]. After i.c. inoculation of 100 pfu of murine coronavirus, all BALB/c mice were highly susceptible and died; in contrast, STS mice were shown to be partially resistant, with a mortality rate of 30%, longer survival times and lower rates of virus production [30]. In our study, RC strain CcS-11, which contains approximately 12.5% genes of the donor strain STS and 87.5% genes of the background strain BALB/c, was more sensitive to TBEV infection than both parental strains BALB/c and STS. The observations of progeny having a phenotype beyond the range of the phenotype of its parents are not rare in traits controlled by multiple genes [18]. Such an observation may be due to multiple gene-gene interactions of quantitative trait loci, which in new combinations of these genes in RC or chromosomal substitution can lead to the appearance of new phenotypes that exceed their range in parental strains [18]. Another possibility is when some progeny receives predominantly susceptible alleles from both parents. These susceptible alleles are distinct from genes *H2*, *Cd209*, *Tlr3*, *Ccr5* and *Oas1b,* which are involved in genetic control of susceptibility to flavivirus encephalitis (see above), because these genes are in CcS-11 localized on segments derived from the background BALB/c strain [Figure 1A and Mouse Genome Informatics (http://www.informatics.jax.org/)]. We also cannot exclude the possibility of a spontaneous mutation appearing during inbreeding and causing the unique phenotype [18]. Similarly to our study, CcS-11 mice were more susceptible to infection with *Leishmania tropica*[18] and to *Trypanosoma brucei brucei*[31] than both parental mouse strains. However, CcS-11 is more resistant to *L. major* than BALB/c [32].

### TBEV replication rate in infected mice is not the main factor influencing survival

We attempted to identify biological markers that could be related to differences in survival rates of the highly susceptible (CcS-11), medium susceptible (BALB/c) and lowly susceptible (STS) mice infected with TBEV. STS mice had very low and delayed viremia when compared with BALB/c and CcS-11 mice after s.c. inoculation. All mice showed brain infection, but lower titers were detected in brains of STS mice, while BALB/c and CcS-11 mice showed higher titers for 3–4 log_10_ pfu. This indicated lower replication rates of the virus in STS mice, but no difference in replication rates in BALB/c and CcS-11 mice. To avoid virus-host interactions before virus neuroinvasion, mice were inoculated i.c., and the dynamics of virus growth in the brain was investigated. Although BALB/c, CcS-11 and STS mice showed different MST after i.c. inoculation, almost the same virus titers were observed in all investigated mouse strains at all intervals p.i. (Figure 2). Virus replication rate is not, therefore, the main factor influencing MST of the host. Similarly, no differences in tissue tropism, viral load or kinetics were observed during acute West Nile virus infection in mouse strains C57BL/6 and C3H/HeN with higher and lower survival rates, respectively [33].

### Higher mRNA levels of cell surface marker of B-cells were seen in brains of the mice less susceptible to TBEV infection

Mice differing in susceptibility to TBEV infection exhibited some differences in immune cells accumulation in brains during the development of encephalitis as demonstrated by quantification of mRNA of cell-specific markers by real-time RT-PCR. In our previous work using BALB/c, SCID on the BALB/c background, C57BL/6 and C57BL/6^*Cd8*−/−^ mice we reported that CD8^+^ T-cells have a dual role and mediate recovery, but also immunopathology during TBE [2]. In this study, no differences in the level of CD8 mRNA were seen between the BALB/c, CcS-11 and STS mice after s.c. inoculation. After i.c. inoculation, only STS mice exhibited slightly higher levels of CD8 mRNA (but also CD4 mRNA) than BALB/c mice at day 6 p.i., but no other differences were observed (Figure 3B). The most interesting results provided quantification of mRNA for CD19, a cell surface marker of B-cells. No increase of CD19 mRNA was observed after i.c. inoculation in all mouse strains. After s.c. inoculation, strains BALB/c and CcS-11 did not increase the levels of CD19 mRNA in their brains, whereas STS mice exhibited a significant increase (Figure 3A). This might indicate stronger B cell infiltration in brains of the less susceptible mice. B cells were also observed in brains in some studies focused on flavivirus encephalitis [34], predominantly found in the perivascular space. The proportion of B and T cells in the brain may vary depending on the infecting flavivirus [35,36]. It is known that TBEV, unlike to Japanese encephalitis and West Nile virus infections, induces CXCL13, a B-cell chemoattractant, in the CNS [37]. Although the antibody reaction is clearly important systemically [38], the importance of B cells infiltrating brain parenchyma in confining of the infection is not clear.

### More severe form of TBE associated with higher expression of proinflammatory cytokines/chemokines in the brain

Accumulation of cytokines and chemokines in the CNS may accentuate progression of encephalitis instead of restricting viral replication [37,39-41]. Deregulated or excess release of proinflammatory mediators by the brain may induce tissue damage with different pathology and disease outcome [42]. In this study, we examined the cytokine/chemokine response in brains of the mice differing in susceptibility to TBEV infection (Figures 4 and 5). We showed that a wide range of cytokine and chemokine mRNAs for TNF-α, IFNγ, IL-1α, IL-1β, IL-2, IL-6, IL-10, CCL2/MCP-1, CCL5/RANTES, IP-10, MIP-1α, MIP-1β and others was produced in response to TBEV infection. There was no clear correlation between the peak of cytokine/chemokine production and the increase of TBEV in the brain. After i.c. inoculation, all mice had comparable virus titers in the CNS (Figure 2C), but the levels of cytokine/chemokine expression differed markedly (Figure 5). STS mice had the highest levels of IL-1β mRNA after i.c. TBEV inoculation when compared to other strains of mice. In contrast, it is known that higher levels of IL-1β are associated with more severe cases of chikungunya [43]. Most of other cytokine/chemokine mRNAs tested were on the lowest level in STS mice in comparison to CcS-11 and/or BALB/c mice. The highest levels of proinflammatory cytokines/chemokines (IFNγ, CCL3/MIP-1α, CCL2/MCP-1, IP-10) (after s.c. and i.c. inoculation) and IL-5 (after s.c. inoculation) were detected in CcS-11 mice, indicating that these cytokines/chemokines can be associated with a more severe form of TBE in our experimental model. On the other hand, the lowest levels of IL-6 and CCL5/RANTES, and also CCL3/MIP-1α and CCL4/MIP-1β mRNAs, were demonstrated in STS mice. These data support the theory that excessive production of proinflammatory mediators could contribute to development of more severe or acute form of TBE. Similarly to our study, higher levels of CCL5/RANTES, CCL3/MIP-1α, CCL4/MIP-1β and IP-10 mRNA were found to be associated with a lethal form of West Nile virus infection in comparison with the non-lethal form [41]. CCL2/MCP-1 and IP-10 mRNA levels correlated well with the susceptibility of the mouse strains to TBEV infection, i.e., CcS-11 mice had the highest levels, BALB/c medium and STS the lowest. These data were confirmed in two independent experiments. Both these chemokines can have an important role in immunopathology during TBE. It has been demonstrated that neutralization of CCL2/MCP-1 leads to increased survival rates in West Nile virus-infected mice [44]. Expression of IP-10 mRNA exhibited the biggest differences between the studied mouse strains. The chemokine IP-10, which is IFN-γ inducible and has the ability to attract activated T cells in the CNS, has also been shown to be a potent neurotoxin [45]. In human TBE patients, IP-10 can be detected in serum as well as in cerebrospinal fluid [46,47]. Using genetically deficient mice and antibody neutralization approaches, it was demonstrated that IP-10 is critical for the recruitment of T-cells into the CNS and survival from West Nile virus infection in mice [48]. However, excessively high levels of IP-10 in the CNS can be very harmful to the host. IP-10 levels in the CNS of HIV-1-infected individuals correlate positively with disease progression. There is also evidence that IP-10 participates in the neuropathogenesis of SHIV-infected macaques [49,50] by contributing to the degeneration of neurons possibly through the activation of a calcium-dependent apoptotic pathway [45,51]. Our data strongly suggest that higher levels of IP-10 in the CNS might be associated with more severe forms of TBE. Further studies using mice specifically knocked out for one of the genes identified will determine their contribution to host susceptibility to TBE. Whether the profile of the chemokine production in human cases of TBE will predict the disease outcome requires further study.

### Key role of neutralizing antibodies in preventing neuroinvasion and host fatality during TBE

The importance of neutralizing antibodies in the prevention of flaviviral diseases is well accepted. As a rule, the antibodies with relatively high affinity and avidity to the surface proteins of TBEV virions can be detected in neutralization test. Only such antibodies interfere with the interaction of the virus with receptors, preventing it to enter the cell. TBEV infection is associated with breakdown of the host blood–brain barrier [26]; therefore, the antibodies can penetrate into the CNS. In our study, STS mice had a strong neutralizing antibody response, CcS-11 mice failed to produce neutralizing antibodies, and BALB/c mice developed neutralizing antibodies but later and at low titers (Figure 6). Therefore, our data on TBE support the importance of neutralizing antibodies in preventing neuroinvasion and host fatality. Similar data were obtained in experiments with the mouse strains C3H/HeN and DBA/2 with higher and lower susceptibility to the Japanese encephalitis virus, respectively [40]. In human TBE patients, those who fail to produce TBEV neutralizing antibodies usually have a very severe course of the infection [52]. The same is seen in patients who receive vaccination against TBEV and develop antibody response, but their antibodies do not have neutralizing capacity [52].

## Conclusions

In summary, our experimental model showed that earlier and greater amounts of neutralizing antibodies could limit neuroinvasion of TBEV and disease progression in less susceptible mice. Moreover, we described that resistant mice exhibit stronger B-cell infiltration (as measured by quantification of changes in mRNA levels of cell surface markers) but lower cytokine/chemokine production in the CNS when compared to the more susceptible mouse strains. The most susceptible mice have the highest overall cytokine/chemokine production in the CNS, particularly of CCL2/MCP-1 and IP-10, which can elevate the primarily proinflammatory environment in the brain, which could result in brain tissue damage and death of the host through immunopathology. This suggests an important role of cytokines and chemokines in the immunopathogenesis of TBE. The host immune response based on the genetic background of the host may therefore play a central role in determining the outcome of TBEV infection.

## Abbreviations

TBE: Tick-borne encephalitis; TBEV: Tick-borne encephalitis virus; s.c: Subcutaneous; i.c: Intracerebral; pfu: Plaque-forming unit; RC: Recombinant congenic; TNF-α: Tumor necrosis factor-α; IFNγ: Interferon γ; IL-1α: Interleukin 1α; IL-1β: Interleukin1β; IL-2: Interleukin 2; IL-4: Interleukin 4; IL-5: Interleukin 5; IL-6: Interleukin 6; IL-10: Interleukin 10; CCL2: (chemokine ligand 2)/MCP-1 (monocyte chemotactic protein-1); CCL3/MIP-1α: Macrophage inflammatory protein-1α; CCL4/MIP-1β: Macrophage inflammatory protein-1β; CCL5/RANTES: Regulated upon activation normal T-cell expressed and secreted; IP10/CXCL/10: Interferon-γ-inducible protein-10.

## Competing interests

The authors declare that they have no competing interests.

## Authors’ contributions

DR conceived and designed the research and wrote the manuscript. MP, JS and JV performed the experiments. MP, JS, JK, LG, ML and PD participated in the design of the experiments and coordination and helped to draft the manuscript. All authors read and approved the final manuscript.

## Supplementary Material

Additional file 1: Figure S1 Differential survival of BALB/c, STS and selected RC strains after subcutaneous inoculation of TBEV. Mice were inoculated with 104 pfu of TBEV and observed for lethality. **Figure S2:** Differential survival of BALB/c, STS and selected RC strains after intracerebral inoculation of TBEV. Mice were inoculated with 10 pfu of TBEV and observed for lethality. Click here for file
