# Supplementary material for: Mice with different susceptibility to tick-borne encephalitis virus infection show selective neutralizing antibody response and inflammatory reaction in the central nervous system
Source: J Neuroinflammation. 2013 Jun 27;10:77. doi: 10.1186/1742-2094-10-77 (PMC3700758; doi:10.1186/1742-2094-10-77)
Supplement: Additional file 1: Figure S1 — Differential survival of BALB/c, STS and selected RC strains after subcutaneous inoculation of TBEV. Mice were inoculated with 104 pfu of TBEV and observed for lethality. Figure S2: Differential survival of BALB/c, STS and selected RC strains after intracerebral inoculation of TBEV. Mice were inoculated with 10 pfu of TBEV and observed for lethality. [file 1742-2094-10-77-S1.pdf]

Supplementary information Fig. 1

A

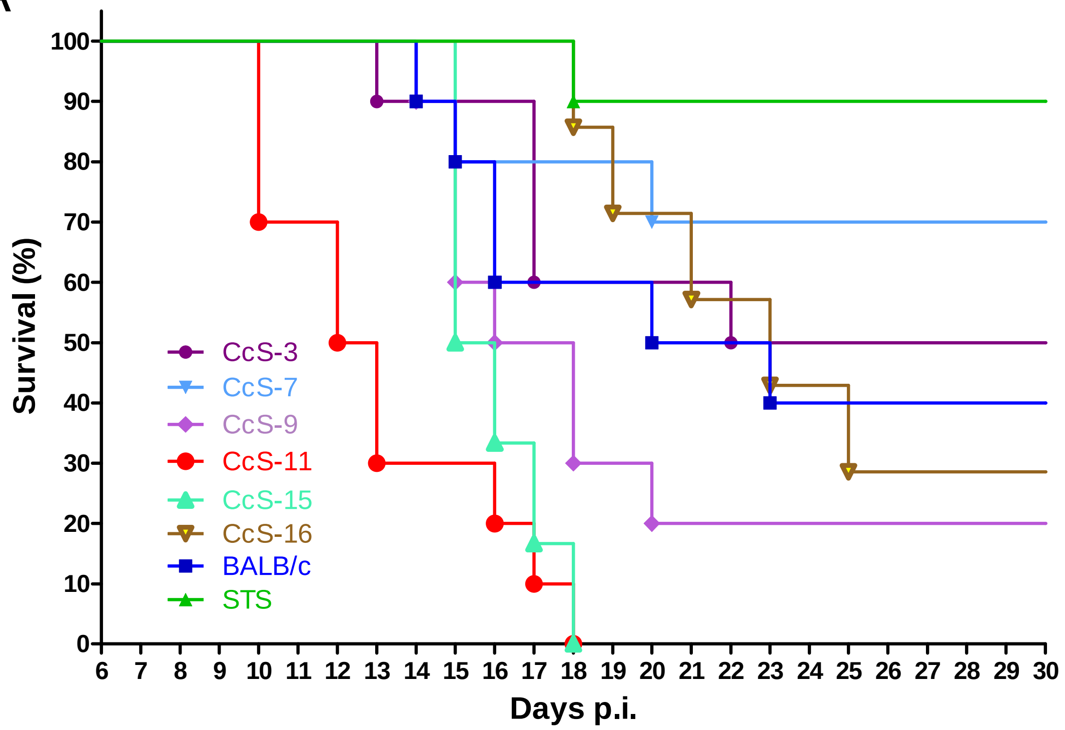

B

Strain	CcS-3	CcS-7	CcS-9	CcS-11	CcS-15	CcS-16	BALB/c	STS
Median survival time [days]	24,1	26,6	19,3	13,1	16,0	24,0	22,8	29,7
Standart deviation [days]	±7,6	±7,2	±6,4	±3,0	±1,3	±5,3	±7,5	±4,1
n survived / n	5/10	7/10	2/10	0/10	0/6	2/7	4/10	9/10

Supplementary Figure 1. **Differential survival of BALB/c, STS and selected RC strains after subcutaneous inoculation of TBEV.** Mice were inoculated with 10^4 pfu of TBEV and observed for lethality.

Supplementary information Fig. 2

A

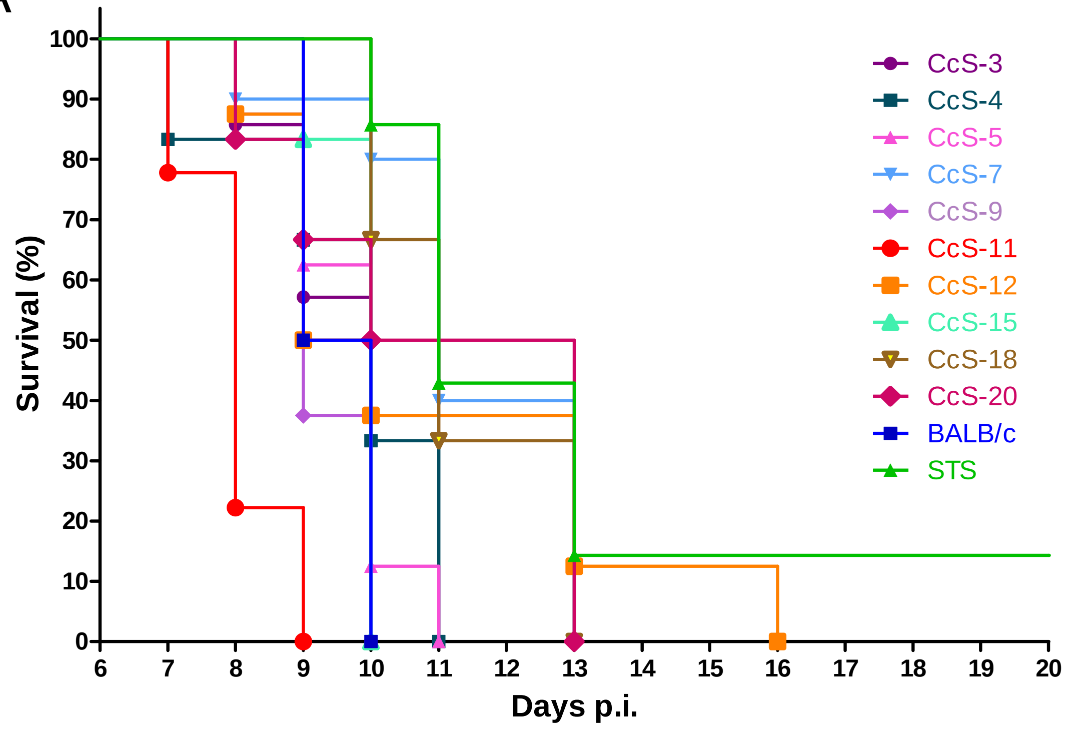

B

Strain	CcS-3	CcS-4	CcS-5	CcS-7	CcS-9	CcS-11	CcS-12	CcS-15	CcS-18	CcS-20	BALB/c	STS
Median survival time [days]	9,4	9,7	9,8	11,4	10,4	8,0	10,9	9,8	11,3	11,0	9,5	12,4
Standart deviation [days]	±0,8	±1,5	±0,7	±1,6	±2,2	±0,7	±2,8	±0,4	±1,4	±2,3	±0,5	±2,7
n survived / n	0/7	0/6	0/8	0/10	0/8	0/9	0/8	0/6	0/6	0/6	0/8	1/7

Supplementary Figure 2. **Differential survival of BALB/c, STS and selected RC strains after intracerebral inoculation of TBEV.** Mice were inoculated with 10 pfu of TBEV and observed for lethality.
